# Zimmermann-Laband-1 Syndrome: Clinical, Histological, and Proteomic Findings of a 3-Year-Old Patient with Hereditary Gingival Fibromatosis

**DOI:** 10.3390/biomedicines7030048

**Published:** 2019-06-29

**Authors:** Federica Guglielmi, Edoardo Staderini, Federica Iavarone, Laura Di Tonno, Patrizia Gallenzi

**Affiliations:** 1 Istituto di Odontoiatria, Fondazione Policlinico Universitario A. Gemelli IRCCS, Università Cattolica del Sacro Cuore, 00168 Rome, Italy; 2 Istituto di Biochimica e Biochimica Clinica, Università Cattolica del Sacro Cuore, Fondazione Policlinico Universitario A. Gemelli, IRCCS, 00168 Rome, Italy

**Keywords:** gingival fibromatosis, Zimmermann-Laband syndrome, periodontal disease, oral microbiome

## Abstract

Background: Zimmermann-Laband-1 syndrome (ZLS-1; OMIM# 135500) is a rare genetic disorder whose oral pathognomonic sign is the development of progressive, diffuse, and severe gingival hypertrophy. Most children with abnormally gingival hyperplasia may also present multiple unerupted teeth and skeletal deformities of maxillary arches (i.e., skeletal anterior open bite). Despite phenotypic variability of the clinical spectrum, gingival fibromatosis is the hallmark of ZLS-1. Method: In this study, we report a 3-year-old male patient with a ZLS-1-related gingival overgrowth and failure of eruption of the deciduous teeth in the molar area. Surgical excision was performed under general anesthesia. Results: At three weeks follow-up, esthetics was significantly improved in terms of gingival appearance, and teeth eruption allowed an adequate masticatory function. Conclusion: In severe cases, surgical removal of the hyperplasic fibrous tissue may be required to expose unerupted teeth and establish a proper gingival contour. Surgical excision under general anesthesia is an elective procedure for patients with special needs, mental disability, as well as young and adult patients with dental anxiety type II and IV associated with poor oral health.

## 1. Introduction

The term “Laband syndrome”, also known as Zimmerman-Laband syndrome (ZLS), indicates an extremely rare genetic disorder characterized by abnormalities of the facial area and the extremities (hands and feet). Most children with ZLS-1 report generalized gingival hyperplasia, thus involving functional and aesthetic issues. Furthermore, affected infants may exhibit abnormally long and thin fingers and toes and/or deformed or absent nails at birth. In some cases, cataract, hepatosplenomegaly, hypertrichosis, hearing loss, and mental retardation may be present. [1]

ZLS-1 is part of a rare and heterogeneous group of disorders called “hereditary gingival fibromatosis”, it affects around 1/1,000,000 patients with both autosomal dominant and recessive inheritance [2]. Clinically, it is associated with local or diffuse non-hemorrhagic gingival enlargement. The underlying alveolar bone is not directly influenced by gingival hyperplasia, but the gingival excess can allow plaque accumulation, causing biofilm-induced gingivitis, pseudopockets, caries, and halitosis. Failure of deciduous and permanent tooth eruption often occurs [3].

We report a case of multidisciplinary management of a hereditary gingival fibromatosis in a 3-year-old male patient who had cutaneous, phenotypic, and molecular features of ZLS-1. The present study is reported according to the CARE (CAse REport) guidelines [4].

## 2. Experimental Section

### 2.1. Patient Information

A 3-year-old child was referred for clinical examination to the Pediatric Dentistry Unit of the Department of Surgical Sciences for Head and Neck Diseases, Agostino Gemelli University Hospital of Rome: during the early diagnostic screening for suspected genetic abnormalities, the pediatricians of the Rare Diseases Unit noticed generalized gingival fibromatosis with poor periodontal health. 

### 2.2. Medical, Family, and Psychosocial History 

The child presented clinically the salient dysmorphogenetic features of ZLS-1: middle-degree intellectual disability with autistic traits, motor impairment with ataxic gait notes, and structural epilepsy. The genetic analysis showed a pathogenetic mutation in the KCNH1 gene on chromosome 1q32.2 (OMIM# 135500) with a heterozygous de novo transmission. The child was born at 42 weeks of gestation regularly from non-consanguineous parents in apparent good health; his mother was born in Italy, but she has Cape Verdean origin. 

**Figure 1:**
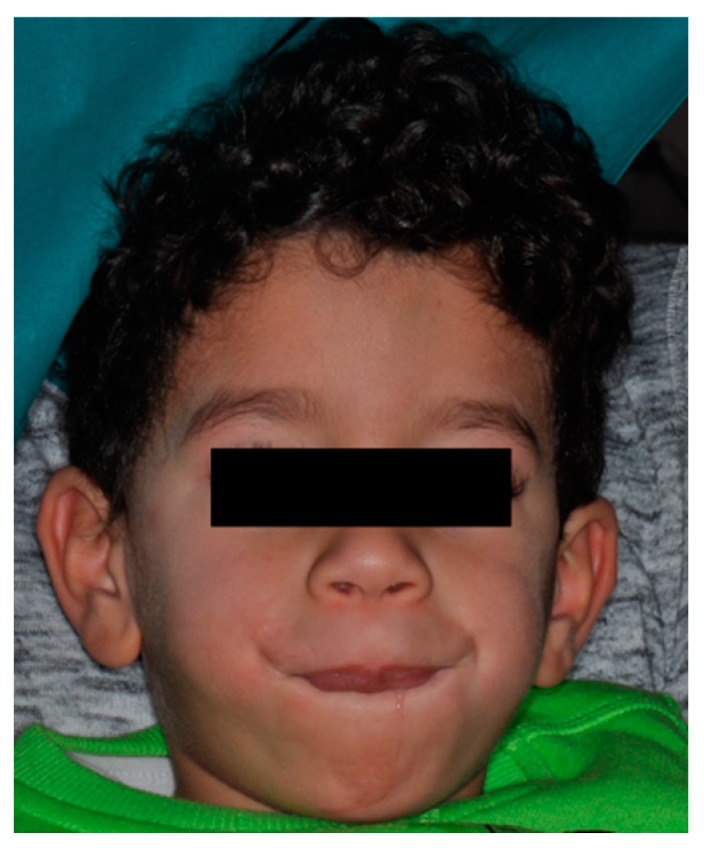
Facial phenotype of the child with Zimmermann-Laband-1 syndrome.

**Figure 2:**
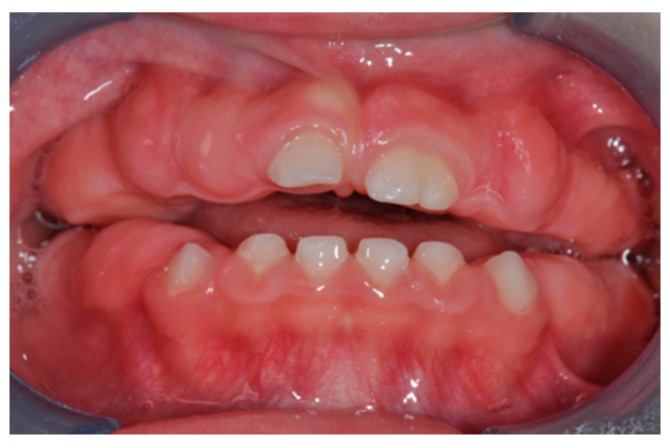
Pre-treatment intraoral photo.

During the first observation, we noticed a delay in dental eruption and a severe generalized hypertrophy of the buccal and lingual aspects of the attached and marginal gingiva; no decays and/or lesions involving oral mucosa were detected. At this time, intraoral photographs were taken (Figure 1 and Figure 2). It was therefore decided, in agreement with the parents, to keep the situation under control with re-evaluation every 6 months. 

### 2.3. Therapeutic Intervention

Two years after the first observation, the child came for a check-up appointment. The parents revealed that the child was diagnosed with Zimmermann-Laband-1 syndrome, associated with a true gingival hyperplasia.

The progressive gingival enlargement, particularly in the molar area, was associated with failure of eruption of the deciduous teeth. Therefore, it was proposed to the parents to remove the gingival overgrowth in order to improve the clinical display of the crowns. Owing to his uncooperative attitude and his medical history, gingival excision was performed under general anesthesia in a day surgery service designed for patients with special needs. The case study was conducted with the parents’ understanding and written consent to the intervention and research participation.

**Figure 3:**
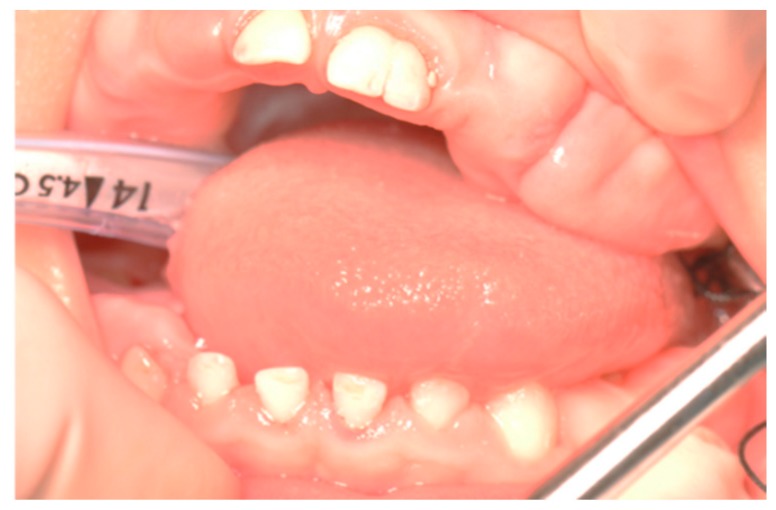
Intra-operative photo: orotracheal intubation in general anesthesia; gingival fibromatosis.

**Figure 4:**
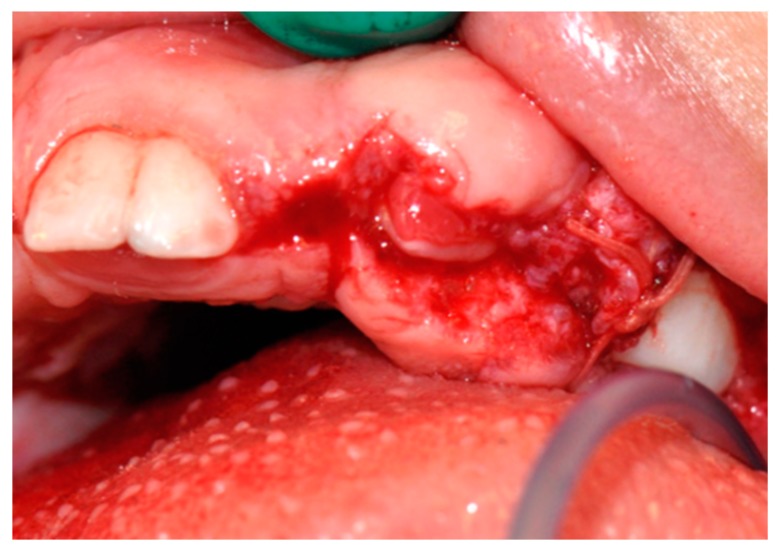
Intra-operative photo: gingivectomy and gingivoplasty surgery.

Using a periodontal probe, the probing pocket depth (PPD) was preoperatively measured on the circumference of the teeth. In order to evaluate the thickness of the connective tissue, a small punch was made on the buccal and palatal aspect of each tooth for which gingivectomy was carried out. Using a 15c blade, a perpendicular paramarginal non-beveled incision was made on the vestibular and palatal aspect. Then, an external intrasulcular incision was carried out to reach the previous horizontal incision, thus delimitating a marginal flap that was sculpted, lifted, and removed. Since the hyperplastic gingiva had a thick keratinized dense fibrous tissue, large-grain and fine-grain olive-shaped diamond burs were used to create a sloping plane projected externally on the vestibular aspect. The external bevel was performed to preserve a proper gum architecture after surgery as well as to allow the apical placement of surgical flap (Figure 3 and Figure 4). An ultrasound tip was used to remove any little fibrous fragment between teeth. The excised gingival tissue was sent for histopathological analysis. 

## 3. Results

**Figure 5:**
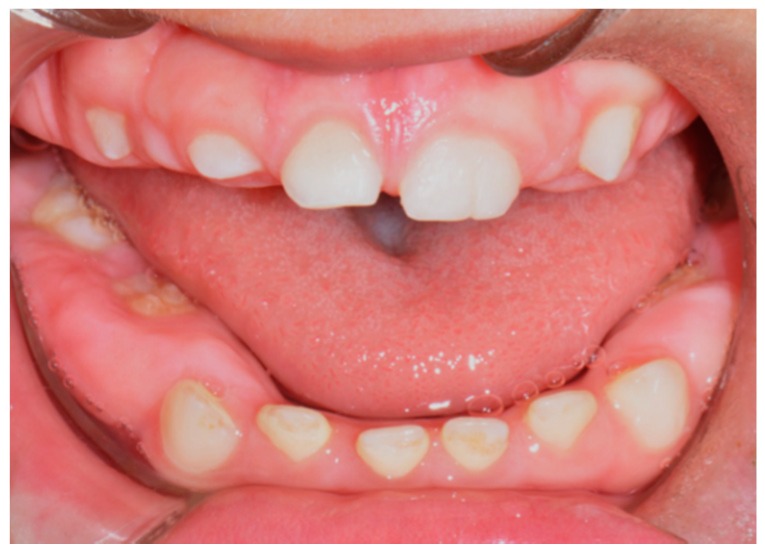
Post-operative photo: soft tissue healing 3 months later.

After three weeks, the patient showed complete healing; then, follow-up appointments were scheduled every three months (Figure 5). Esthetics was significantly improved in terms of gingival appearance, and teeth eruption allowed an adequate masticatory function. 

### 3.1. Histological Characteristics

**Figure 6:**
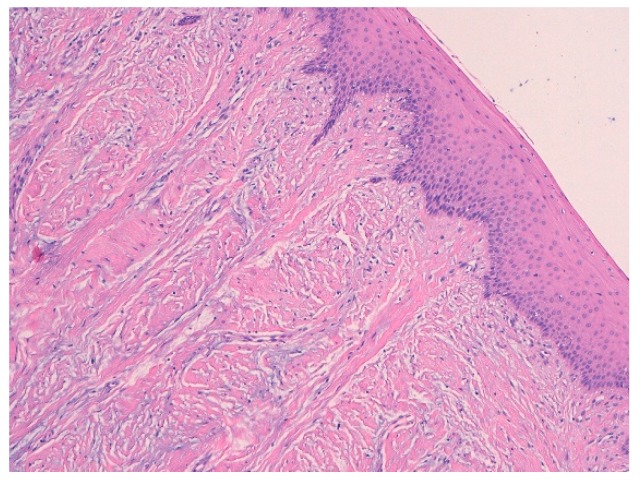
Histological findings of the biopsied sample: the squamous-lining oral mucosa with parakeratosis and pseudoepitheliomatous hyperplasia.

**Figure 7:**
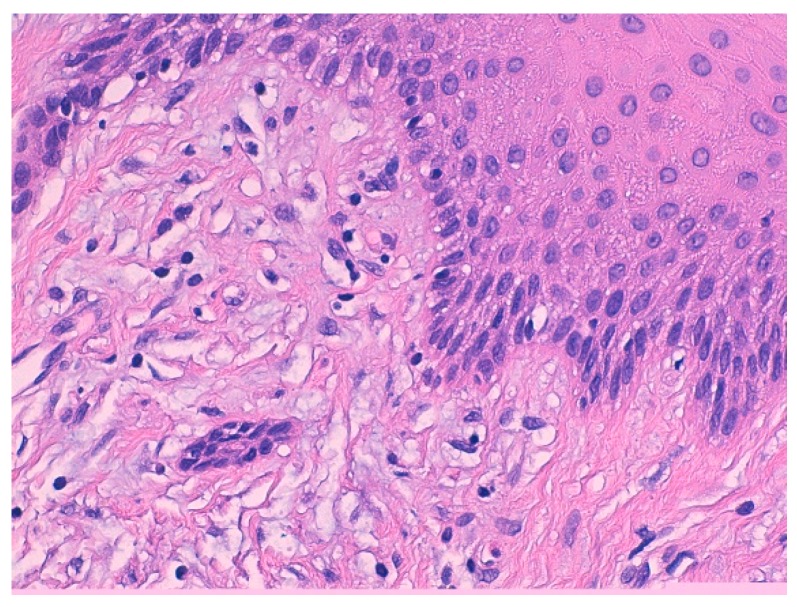
Histological findings of the biopsied sample: the subepithelial deposition of dense hypocellular connective tissue collagenized with focal mixoidal changes.

Histological features of the biopsied sample revealed the presence of squamous-lining oral mucosa with parakeratosis and pseudoepitheliomatous hyperplasia along with the subepithelial deposition of dense hypocellular connective tissue collagenized with focal mixoidal changes. No obvious atypia was observed (Figure 6 and Figure 7).

### 3.2. Analysis of Salivary Biomarkers

The unstimulated saliva was collected following a standardized protocol [5]. The patient had fasted for at least two hours before the appointment. Saliva was collected in the morning between 10:00 A.M. and 12:00 P.M. 

Saliva was subsequently moved to a plastic container in an ice bath. Afterwards, it was combined with an acidic solution (0.2% 2,2,2-trifluoroacetic acid (TFA)) in 1:1 *v*/*v* ratio. The sample was subjected to a centrifugation at 8000 g for 10 min at 4 °C. Finally, the acidic supernatant was separated from the precipitate and stored at −80 °C until the analysis was carried out by HPLC-ESI-MS (100 /l, corresponding to 50/ l of saliva). The whole salivary sample of the child was analyzed through a top-down proteomic approach: intact protein ions or large protein fragments were subjected to gas-phase fragmentation for MS analysis [6,7]. A preliminary analysis showed an increase in inflammatory state proteins compared to healthy subjects of the same sex and age; moreover, the defensins alpha 1 and alpha 3 were doubled compared to the controls, and the presence of the S100A7 was also highlighted.

## 4. Discussion

Clinical follow-ups were scheduled every 6 months because the recurrence risk of the gingival fibromatosis is reported to be high for 6-year follow-up.

Based on the histopathological, clinical, and molecular findings, a differential diagnosis of ZLS-1 was made excluding the other types of hereditary gingival fibromatosis (HGF). (Figure 7, Table 1)

The analysis of salivary biomarkers suggests that the salivary concentration of inflammatory markers in patients with gingival fibromatosis is upregulated in comparison to that found in healthy patients. Several articles have recently outlined the potential use of saliva as a diagnostic fluid. Saliva is a non-invasive and indispensable source of information for diagnosis of rare diseases. Recent proteomic platforms have analyzed the human salivary proteome and characterized about 3000 differentially expressed proteins and peptides. Future investigations are needed to identify chronic inflammatory proteins and molecular markers for genotype-dental phenotype correlation of ZLS.

## 5. Conclusions

The aim of this study was to present the multidisciplinary management of a syndromic child with hereditary gingival fibromatosis, showing that a team of geneticists, dentists, pathologists, and biochemists can work together to achieve a comprehensive diagnosis and treatment of rare diseases [8]. In severe cases, surgical removal of the hyperplasic fibrous tissue may be required to expose unerupted teeth and establish a proper gingival contour. Surgical excision under general anesthesia is an elective procedure for patients with special needs, mental disability, as well as young and adult patients with dental anxiety type II and IV associated with poor oral health [9,10].

## Figures and Tables

**Table 1:** Classification of hereditary gingival fibromatosis.

Classification of Hereditary Gingival Fibromatosis
1. Hereditary gingival fibromatosis
2. Gingival fibromatosis with craniofacial dysmorphism
3. Gingival fibromatosis with progressive deafness
4. Gingival fibromatosis/ hypertrichosis syndrome
5. Ramon syndrome
6. Zimmermann-Laband syndrome
7. Infantile systemic hyalinosis
8. Juvenile hyaline fibromatosis
9. Oculodental syndrome, Rutherfurd type
10. Amelogenesis imperfecta/nephrocalcinosis syndrome
11. Amelogenesis imperfecta/gingival fibromatosis syndrome
